# A Comparative Study of Isobaric 1% Chloroprocaine With 0.5% Isobaric Bupivacaine for Ureteroscopic Lithotripsy Under Spinal Anaesthesia: A Prospective Randomised Study

**DOI:** 10.7759/cureus.24633

**Published:** 2022-04-30

**Authors:** Sugumar. M, Atul k Singh, Amrita Rath, Reena ., Abhinay Jayanthi

**Affiliations:** 1 Anesthesia and Critical Care, Institute of Medical Sciences, Banaras Hindu University, Varanasi, IND; 2 Anesthesiology and Critical Care, Institute of Medical Sciences, Banaras Hindu University, Varanasi, IND

**Keywords:** motor blockade, sensory blockade, spinal anaesthesia, isobaric, bupivacaine, chloroprocaine

## Abstract

Introduction

Day-care surgery has become an immensely popular modality of treatment throughout the globe. Ureteroscopic lithotripsy (URSL) surgery is commonly performed on a day-care basis, and the duration of surgery ranges from 15 minutes to 45 minutes. URSL is routinely done under spinal anaesthesia. Spinal anaesthesia with conventional drugs has its problems like delayed regression and urinary retention, necessitating a longer hospital stay, thereby increasing the time and resources needed. For day-care surgery, short-acting local anaesthetics have a quicker onset with fewer side effects, and drugs that ensure quicker hospital discharge are more often preferred over long-acting anaesthetics. For spinal anaesthesia, we planned to perform a randomised prospective study to compare the effectiveness of 40 mg of preservative-free 1% chloroprocaine over 10 mg of plain 0.5% isobaric bupivacaine.

Materials and method

After obtaining clearance from the institute’s ethical committee and written informed consent from the patients, 64 patients between the ages of 18 and 50 years of either sex belonging to American Society of Anesthesiologists (ASA) 1 and 2 scheduled for URSL surgery were included in our study. They were randomised using a computer-generated sealed envelope technique into two groups. Group C received 4 ml of 1% isobaric chloroprocaine, and group B received 2 ml of 0.5% isobaric bupivacaine in the intrathecal space. The primary outcome was to compare the in-hospital time among both groups. Our secondary outcomes were the onset time of motor and sensory blocks, the duration of the blocks, time to unaided ambulation and voiding, the need for an overnight stay, and the side effects like postoperative nausea and vomiting (PONV), and urinary retention. The data were analysed using the unpaired t-test and chi-square test and calculated by SPSS 20.0 software version (IBM Corp., Armonk, NY).

Results

Final analyses were done among 60 patients. In-hospital time was significantly lower in group C as compared to group B (p<0.05). The onset time of sensory and motor blockade was significantly lower in group C as compared to group B (p<0.005). The duration of sensory and motor blockade was significantly less in group C as compared to group B (p<0.005). The time for unaided voiding and ambulation was less in group C as compared to group B. The need for an overnight stay was only needed in group B. The incidence of PONV and urinary retention was higher in group B.

Conclusion

In URSL surgery, the use of intrathecal 1% isobaric chloroprocaine 40 mg resulted in a reduced hospital stay time as compared to the use of intrathecal 10 mg of 0.5% isobaric bupivacaine. Also, it resulted in a significantly faster onset and faster regression of the block, less duration of the blockade, shorter time to ambulation and micturition, and a requirement for an overnight stay when compared with isobaric spinal bupivacaine.

## Introduction

Day-care surgery has become an immensely popular modality of treatment throughout the globe. It refers to the practise of admitting and preparing selected patients on the day of surgery, planning for a non-emergency surgical procedure, and their discharge within 24 hours of that surgery [1]. Ambulatory anaesthesia offers various advantages, like reduced risk of nosocomial infections, cost reduction, and a short duration of hospital stay [2]. Spinal anaesthesia is the most convenient anaesthetic technique and is commonly used in surgeries below the umbilicus [3]. Its advantages over general anaesthesia are reduced stress response, rapidity in onset, post-operative pain relief, shorter hospital stay, and cost-effectiveness [4].

Spinal anaesthesia is generally performed by using local anaesthetic drugs at different doses and baricity with or without the addition of an adjuvant. The ideal anaesthetic for spinal anaesthesia in an ambulatory surgery patient would provide a rapid onset of action, adequate potency, predictable (short) duration, and a low incidence of transient neurological symptoms (TNS) and systemic side effects [5].

Bupivacaine is one of the most commonly used local anaesthetic drugs for spinal anaesthesia. Isobaric chloroprocaine provided a shorter sensory and motor block duration with better haemodynamic stability than bupivacaine, which is a desirable feature for early ambulation, voiding, and physiotherapy [6].

Very little literature is available comparing isobaric chloroprocaine's effectiveness with bupivacaine in urological procedures. Ureteroscopic lithotripsy (URSL) surgery is commonly performed on a daycare basis, and the duration of surgery ranges from 15 minutes to 45 minutes [7]. The dose of 1% chloroprocaine used in lower limb surgery was around 40 mg [8]. So, we planned a randomised prospective, double-blind control study to compare the effectiveness of 40 mg of preservative-free 1% chloroprocaine over 10 mg of plain 0.5% isobaric bupivacaine for spinal anaesthesia.

## Materials and methods

After obtaining approval from the Institutional Ethical Committee and written informed consent from the patients, 64 patients of American Society of Anaesthesiologists (ASA) physical status 1 and 2, aged between 18 and 50 years, undergoing elective URSL were included in the study. Patients who had contraindications to spinal anaesthesia, including patient refusal, use of anticoagulants, ASA physical status III-V, lactating or pregnant women, known cases of hypersensitivity/allergy to any of the study medications, and psychiatric/neurological diseases were excluded from the study. Those patients in whom spinal anaesthesia could not be administered because of technical issues or in whom it failed were dropped from the study. The patients were randomly allocated by the sealed envelope method into two groups of 32 patients in each group.

Group C - Patient group received 4 ml (40 mg) of “isobaric 1% Chloroprocaine”

Group B - Patient group received 2 ml (10 mg) of “isobaric 0.5% Bupivacaine’’

The primary outcome was to compare the in-hospital time among both groups. The onset of sensory and motor blocks, the duration of sensory and motor blocks, time to independent micturition, time taken for unaided ambulation, and the need for an overnight hospital stay were our secondary outcomes. The various side effects of the study drugs used were also noted.

All the patients were premedicated with tablets of Ranitidine 150 mg, Metoclopramide 10 mg, and Alprazolam 0.25 mg as per our institutional protocol. After arriving in the operating room, an 18/16G iv cannula was placed, and patients were preloaded with 500 ml of intravenous (iv) crystalloids. In the operating room, baseline readings of heart rate (HR), mean arterial pressure (MAP), and oxygen saturation (SpO_2_) were being recorded by the nurse unrelated to the study.

Under strict aseptic precautions and with the patient lying in a lateral decubitus position, a 25G Quincke (Spinocan, Braun, Meslungen, Germany) spinal needle was inserted into the L4-L5 space. After ensuring a free flow of cerebrospinal fluid (CSF) and negative aspiration of blood, the study drug was administered by an anaesthetist not involved in the study at the rate of 0.2 ml per second as per the allocated group. Intrathecal injections were performed over 30 seconds in both groups. Patients were placed supine with no elevation of the extremities after the injection and were tested every 1.5 minutes until the sensory blockade of L1 was achieved. Once the effect was achieved to the required level, the patient was put in the lithotomy position. HR, MAP, and peripheral SpO_2_ were monitored continuously and values were recorded at a gap of 15 minutes up to the first 60 minutes after administering spinal anaesthesia.

Patients were asked about the pinprick sensation on each side for the purpose of assessing sensory blockade. The Modified Bromage scale was used to assess the motor blockade, where 0 indicates no block; 1 indicates impaired movement at the hip, normal knee, and ankle movements; 2 indicates impaired movement at the hip and knee, but normal ankle movements; and 3 indicates impaired movement at the hip, knee, and ankle.

The in-hospital time was defined as the time from spinal anaesthesia to the time of discharge. Onset time for the sensory block was defined as the time between injection and no sensation at the T10 level. The duration of sensory blockade was defined as the time between injection and recovery of sensation at the S2 dermatome level. Onset time for the motor block was defined as the time between injection and modified Bromage of >2. Motor blockade duration was defined as the period between injection and complete recovery of the motor block (a modified Bromage score of 0).

Failure of the block was defined as the block being attempted but no block ensued or if the block was present but inadequate for the surgery [9]. During surgery, any episode of hypotension (MAP decreased to less than 20% of the baseline value) was treated with intravenous fluids like Ringer’s lactate and an injection of mephentermine iv. Bradycardia (HR < 40 beats/min) was treated with 0.01 mg/kg of injection atropine iv. Patients experiencing desaturation (SpO_2_ < 92%) received oxygen through a simple facemask at 5 L/min.

Post-operatively, patients were transferred to the post-anaesthesia care unit (PACU). The onset time and duration of sensory and motor blockade were recorded in the operation room and post-operative care unit, respectively, by a physician unrelated to the study. Time to first unaided ambulation and time to the first independent micturition were recorded in the PACU by a nurse unrelated to the study. Also, the number of patients with post-operative pain, defined as the visual analogue scale (VAS > 4), and the number of patients who needed an overnight hospital stay were noted.

The same PACU nurse recorded recovery room complications such as post-operative nausea and vomiting (PONV) and post-operative urinary retention. For those who complained of nausea and vomiting, an injection of Ondansetron 4 mg was given intravenously. Pain experienced in the ward was managed with an injection of paracetamol 1 g when VAS > 4.

The categorical data of the demographics profile were analysed by chi-square test and non-categorical data using an unpaired T-test. Statistical analysis was performed using the computer programme SPSS (version 10, SPSS, Inc., Chicago, IL). Analyses of all-time comparisons (onset and duration of motor and sensory blockade, time to first urination, and ambulation) were performed using an unpaired t-test. Changes in MAP were compared using analysis of variance for repeated measures. A p-value of less than 0.05 was defined as statistically significant.

## Results

In group B, out of 32 patients, one patient was dropped out because of a failed spinal anaesthesia, and another patient was dropped out because general anaesthesia was administered intra-operatively because of the long duration of surgery. In group C, however, two patients were dropped due to spinal failure. Therefore, the final analysis was carried out on 30 patients in each group. The baseline characteristics were comparable among both groups (Table 1).

**Table 1:** Baseline characteristics of both groups. Values are in mean ± standard deviation (SD). BMI: body mass index, M: male, F: females, SA: spinal anaesthesia.

Parameters	Group C	Group B	p-value
Age (years)	31.2 ± 7.53	32.2 ± 5.55	0.56
Weight (kilogram)	58.3 ± 3.75	59.14 ± 4.34	0.42
Height (meters)	1.609 ± 0.036	1.608 ± 0.036	0.94
BMI (kg/m^2^)	22.03 ± 1.40	22.47 ± 2.14	0.35
Sex (M/F)	17/13	18/12	0.187
Duration of surgery (minutes)	48.9 ± 4.77	47.13 ± 3.35	0.10
Time from SA to start of surgery (minutes)	10.6 ± 0.88	11.10 ± 1.02	0.06

In-hospital time was significantly lower in group C than in group B (p<0.05; Table 2).

**Table 2:** Comparison of in-hospital time among both groups. Values are in mean ± standard deviation (SD). *p-value < 0.05 is considered statistically significant.

Group	In-hospital time (hours)	p-value*
Group C	3.516667 ± 0.40	0.0001 (<0.05)
Group B	7.36 ± 0.58	

The mean onset time of sensory blockade and of motor blockade was significantly less in group C compared to group B (p<0.05; Table 3). The mean duration time of sensory blockade as well as of motor blockade was significantly lower in group C compared to group B (p<0.0001). The time is taken for independent micturition and first unaided ambulation was significantly lower in group C as compared to group B (p<0.005; Table 3).

**Table 3:** Characteristics of sensory and motor blockade between the groups. Values are in mean ± standard deviation (SD). *p-value <0.05 is considered statistically significant.

Parameters	Group C	Group B	p-value*
The onset of sensory blockade (minutes)	5.066 ± 0.82	6.24 ± 1.07	0.0002
The onset of motor blockade (minutes)	6.833 ± 0.83	8.84 ± 0.84	0.0001
Duration of sensory blockade (minutes)	66.8 ± 4.69	191.5 ± 8.72	0.0001
Duration of motor blockade (minutes)	64.6 ± 5.88	175.33 ± 9.09	0.0008
Time to independent micturition(hrs)	2.84 ± 0.591	5.53 ± 0.597	0.0008
Time taken for unaided ambulation (hrs)	3.516 ± 0.404	7.36 ± 0.58	0.0001

The number of patients in group B requiring an overnight hospital stay post-operatively was statistically significant in group C as compared to group B (p<0.005). None of the patients in group C stayed in the hospital overnight, as compared to 10 patients in group B (Table 4).

**Table 4:** Comparison of post-operatively need for overnight hospital stay between the groups. n means in numbers. p-value <0.05 is considered statistically significant.

Parameter	Group C		Group B		P-value
Overnight hospital stays	Yes (n)	No (n)	Yes (n)	No (n)	0.0005
	0	30	10	20	

The incidence of post-operative nausea, vomiting, and pain was comparable among both groups (p>0.05). However, group C had significantly lower incidences of post-operative urinary retention as compared to group B (Table 5).

**Table 5:** Side effects profile among both groups. n means in numbers. VAS: visual analogue scale. *p<0.05 is considered statistically significant.

Side effects	Group C		Group B		P-value
Post-operative nausea and vomiting	Yes (n)	No (n)	Yes (n)	No (n)	0.31
	0	30	1	29	
Post-operative pain (VAS>4)	3	27	0	30	0.07
Post-operative urinary retention	0	30	10	20	0.0005*

## Discussion

We conducted the study on sixty-four ASA I-II patients aged between 18 and 50 years of either sex who underwent an elective URSL procedure that was expected to be completed within 40 minutes from the time of administering the subarachnoid block. All patients were comparable with respect to age, sex, weight, and height. Baricity being a major determinant of block height, both the study drugs chosen were similar in baricity, i.e., isobaric [10].

In our study, the equivalent doses of chloroprocaine and bupivacaine were used. A study conducted by Kouri and Kopacz found that 40 to 60 mg of 2-chloroprocaine was needed for a reliable sensory block and motor block for brief surgical procedures [11]. Another study found 40 mg of chlorprocaine to be the optimal dose for spinal anaesthesia. They also found that a lower dose resulted in an inadequate duration of anaesthesia and a higher dose only resulted in a prolonged block recovery [12]. In a study by Hejtmanek and Pollock, they found that 10 mg of intrathecal bupivacaine was the optimal dose for daycare surgeries [13]. Therefore, we used the minimum dose that was believed to be clinically efficacious. Regarding volume, previous studies have shown that the dose of the isobaric bupivacaine solution, and not the volume, determines the intensity and duration of sensory and motor blockade [14-16]. Therefore, we administered 10 mg of isobaric bupivacaine as a 2 ml solution in its original marketed formulation.

In group C, in one patient, the plan of anaesthesia was converted to general anaesthesia because the surgical time exceeded more than 70 minutes and the patient complained of discomfort. Block failure is frequently attributed to one of three factors: clinical technique, inexperience (particularly among unsupervised trainees), and a failure to recognise the importance of a meticulous approach [17]. The reason for the failed spinal in our three patients can be any one of the above-proposed mechanisms.

The mean in-hospital stay time is reduced in the chloroprocaine group, which is highly desirable in daycare procedures like URSL. It not only reduces the burden on hospital staff and hospital resources but also becomes cost-effective, time-saving, and comfortable on the part of the patient. The mean onset time of the study drug to attain sensory block at the L1 dermatomal level and motor blockade (modified Bromage > 2) was shorter in the chloroprocaine group than in the bupivacaine group, which is supported by the previous literature [18-20]. The mean offset time of sensory block to S2 level and motor block (modified Bromage of 0) was shorter in the chloroprocaine group than in the bupivacaine group. These findings are consistent with the previous literature, making it a safer alternative to longer-acting drugs for spinal anaesthesia in daycare surgical procedures [21-23].

**Figure 1:**
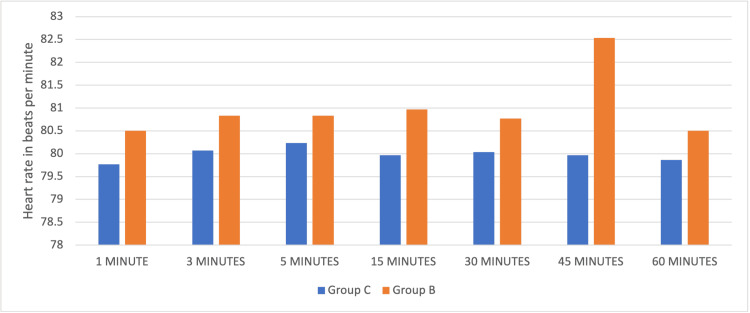
Comparison of intra-operative heart rate at various intervals among both groups.

**Figure 2:**
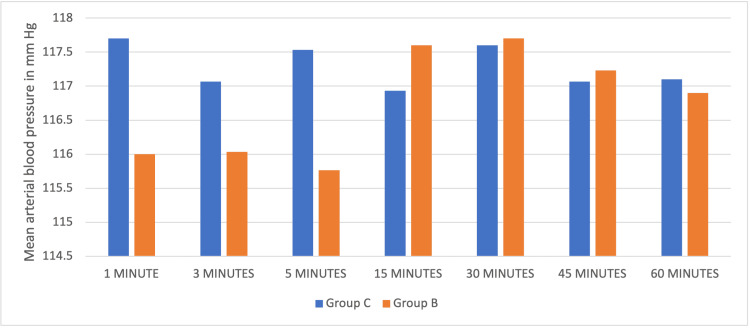
Comparison of intra-operative mean arterial blood pressure at various intervals among both groups.

Haemodynamics during the intra-operative period was comparable between both groups (Figures 1-2). There were no incidents of hypotension requiring vasopressors or bradycardia in either group for the dose used.

Three patients in the chloroprocaine group experienced mild to moderate pain in the recovery room and iv paracetamol was given as rescue analgesia, whereas none of the patients in group B needed rescue analgesia. This can be explained because spinal anaesthesia regressed more rapidly in group C than in group B. However, the pain was easily controlled with non-opioid-based analgesics.

The mean time for unassisted ambulation and unaided voiding was faster with the chloroprocaine group than in the bupivacaine group, which makes it a better choice for a daycare short procedure. This delay in unaided voiding may be explained by the fact that regression of sensory block up to S3 is needed for normal detrusor muscle contraction during the process of voiding. A similar finding was also found in the previously published literature [24].

Urinary retention following spinal anaesthesia for lower limb procedures is well documented in the literature, and the cited rate of post-operative urinary retention varies in the literature from 5% to 70% [24]. None of the patients in group C complained of any urinary retention. However, 10 patients in group B complained of urinary retention and needed urinary catheterization, necessitating an overnight hospital stay. The main principle behind daycare surgery is to minimise the duration of hospital stays. Therefore, it is very evident that chloroprocaine is a better choice in terms of the duration of hospital stay for a daycare procedure. One patient in group B complained of PONV and was managed with iv ondansetron and was kept overnight for observation.

Our study has a few limitations too. The volumes of drugs used in our study were different. The observer bias was minimised by data collection by a physician blinded to group allocation and who was not present during spinal drug administration.

Though in common practice, we did not use adjuncts to local anaesthetics which could have prolonged the analgesia, because that would have interfered with our study results. More studies can be conducted using adjuncts to local anaesthetics in the future.

## Conclusions

Our study concludes that 1% isobaric chloroprocaine is a better alternative to isobaric bupivacaine for daycare surgery like URL because of the reduced need for an in-hospital stay. Also, the quicker onset and offset times when used intrathecally make it a better choice in URSL. The incidence of side effects like urinary retention and PONV was much lower in the chloroprocaine group than in the bupivacaine group. Urinary retention, which is a common post-operative problem following spinal anaesthesia, was absent in the chloroprocaine group, and thus, quicker hospital discharge makes it a good choice for daycare procedures.
